# Pulmonary Peripheral Carcinoids after Diffuse Idiopathic Pulmonary Neuroendocrine Cell Hyperplasia and Tumorlets: Report of 3 Cases

**DOI:** 10.1155/2015/851046

**Published:** 2015-11-03

**Authors:** Carlos Abrantes, Rui Caetano Oliveira, Joana Saraiva, João Bernardo, Lina Carvalho

**Affiliations:** ^1^Department of Anatomical Pathology, Hospital of the University of Coimbra (CHUC), Portugal; ^2^Department of Anatomical Pathology, Portuguese Oncology Institute of Coimbra (IPOC), Portugal; ^3^Department of Thoracic Surgery, Hospital of the University of Coimbra (CHUC), Portugal; ^4^Institute of Anatomical Pathology and Molecular Pathology of the Medical Faculty of University of Coimbra (IAP-FMUC), Portugal

## Abstract

Diffuse idiopathic pulmonary neuroendocrine cell hyperplasia (DIPNECH) and tumorlets are neuroendocrine cells proliferations smaller than 5 mm. The former confines to bronchial/bronchiolar wall, while the latter broke through epithelial basement membrane. The authors present 3 cases of DIPNECH and tumorlets associated with a typical peripheral carcinoid tumor without underlying lung disease. The patients presented with nonspecific pulmonary symptoms: 3 females, 60, 72, and 84 years old, whose CT-scans showed well-defined pulmonary nodules, 2.2, 1.6, and 1.4 cm, respectively; first patient was submitted to lobectomy and the others underwent surgical biopsy. Whitish/brownish lobulated tumors corresponded to typical carcinoids (less than 2 mitoses/2 mm^2^ and without necrosis); polygonal/elongated cells under lobular pattern expressed CD56, chromogranin A, synaptophysin, and CK7; Ki-67 positivity was between 1 and 3%. Bronchial/bronchiolar wall neuroendocrine cell hyperplasia and several neuroendocrine nodules under 5 mm, with identical morphologic and immunoexpression, were observed, without lung disease. Typical carcinoid associated with DIPNECH and tumorlets without other pulmonary diseases is rare. Sporadic cases may recall embryonal neuroendocrine differentiation potentiality to develop peripheral hyperplasia, most commonly in underoxygenated parenchyma. The described cases are elucidative of peripheral spectrum of neuroendocrine cell tumour evolution, reinforcing higher female incidence as in central carcinoids, still without a clear preneoplastic lesion.

## 1. Introduction

Adult lung retains pools of neuroendocrine (NE) cells, located in upper airways till the terminal respiratory unit, related to embryonic development. These specialized epithelial cells receive neural impulses and secrete several hormonally active substances, mainly under reduced oxygen tension [1].

Reactive NE cell hyperplasia is then commonly seen after airway fibrosis and/or inflammation, associated with evident distortion of the lung microarchitecture and hypoxia [1, 2]. However, a rare and underrecognized disorder called diffuse idiopathic pulmonary neuroendocrine cell hyperplasia (DIPNECH), named by Aguayo et al. [3], is defined as diffuse NE cell hyperplasia confined to the respiratory epithelium layer without penetration of the basement membrane and not associated with any known predisposing condition. The proliferating NE cells may release amines and peptides which induce secondary mild inflammation and fibrosis in the airways [4], alterations frequently seen on histopathological examinations of these cases and probably responsible for the asthma-like presentation in symptomatic patients.

DIPNECH is considered to be a precursor of tumorlets and carcinoid tumors [1, 2, 4], where well-differentiated NE cell proliferation has broken through epithelial basement membrane, with less than 5 mm in diameter for tumorlets and above 5 mm for carcinoid tumors [4].

Carcinoid tumors are reported to represent less than 1% of all lung malignant tumors [2, 4] and are classified by the World Health Organization (WHO) into typical with fewer than 2 mitoses per 2 mm^2^ and absence of necrosis (low grade epithelial malignancy and 70 to 90% of cases) or into atypical, when 2–10 mitoses per 2 mm^2^ and/or foci of necrosis are seen (intermediate grade epithelial malignancy and 10 to 30% of cases) [4].

The aim of this small series is to describe 3 rare cases where the association and progression from DIPNECH to tumorlets and carcinoid tumors are evident and to perform a small review of the literature.

## 2. Materials and Methods

Between 1989 and 2013, 158 carcinoid tumors were diagnosed at* Centro Hospitalar e Universitário de Coimbra* (CHUC) [5], a tertiary and teaching hospital, serving as a reference to three million inhabitants in Portugal. Of these, 96 corresponded to typical and 27 to atypical carcinoids; the remaining cases could not be definitively graded due to the characteristics of the available material (mainly small biopsies).

We describe 3 rare cases of DIPNECH associated with tumorlets and carcinoid tumors diagnosed at CHUC, with focus on pathological features, included in the referred casuistic.

Haematoxylin-Eosin (HE) and immunohistochemistry slides were observed under a light microscope Nikon Eclipse 50i and images were obtained using a Nikon-Digital Sight DS-Fi1 camera. The characteristics of applied antibodies are summarized in Table 1.

## 3. Case Reports

### 3.1. Case 1

#### 3.1.1. Clinical Data

A 60-year-old female patient presented with a 2-month history of hemoptysis. There were no alterations in the physical examination or in pulmonary function tests. Computerized Tomography- (CT-) scan of the thorax showed a left lower lobe peripheral nodule with 2.2 cm; there were no signs of interstitial lung disease. A diagnosis of probable neuroendocrine carcinoma of the lung was given on a CT-guided core needle biopsy performed at another institution. The patient was then referred to our institution and underwent pulmonary lobectomy with mediastinal lymphadenectomy.

#### 3.1.2. Macroscopy

Macroscopic examination of the lobectomy surgical specimen showed a brownish, lobulated, and well-defined tumor, located close to the pleural surface, which was 2.2 cm. The pulmonary parenchyma had unremarkable macroscopic appearance. Beyond the total paraffin inclusion of the described nodule, representative samples of the pulmonary parenchyma were also included.

#### 3.1.3. Microscopy and Immunohistochemistry

**Figure 1:**
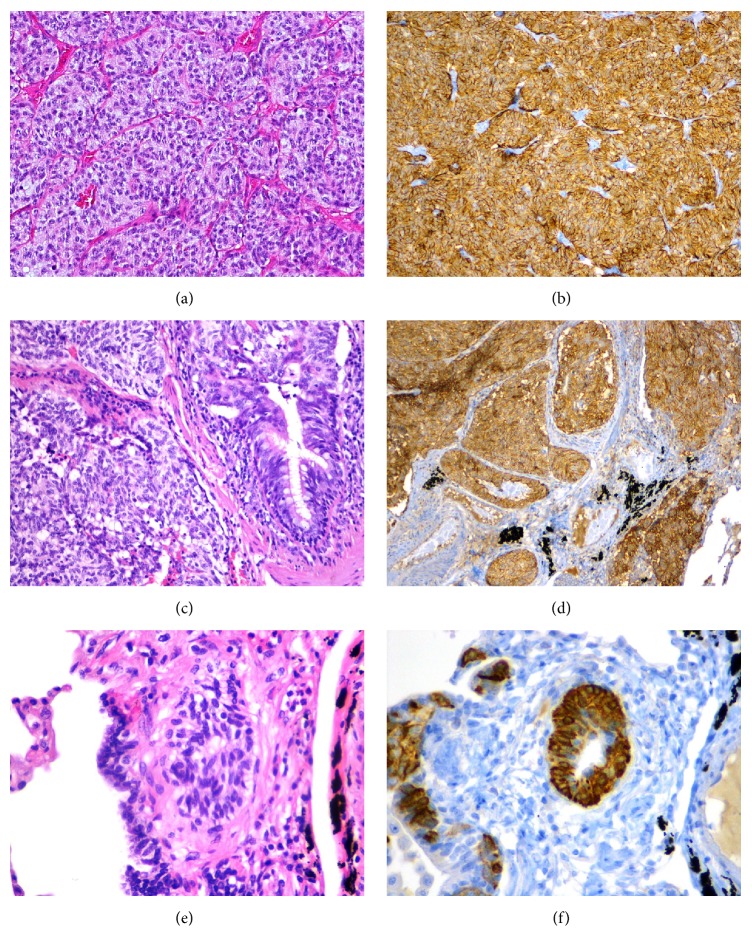
Case 1. (a) Carcinoid tumor. (b) Carcinoid with strong and diffuse expression of CD56. (c) Tumorlet. (d) Tumorlet also showing strong and diffuse expression of CD56. (e) DIPNECH. (f) Strong expression of chromogranin A in DIPNECH.

Microscopic study (Figure 1) showed that the tumor corresponded to a well-differentiated NE neoplasm, with an organoid or trabecular pattern; the cells were polygonal or elongated with round or oval nuclei with “salt and pepper” chromatin and eosinophilic cytoplasm. There were less than 2 mitoses per 2 mm^2^ and necrosis was absent. Immunohistochemistry study showed diffuse staining for cluster of differentiation (CD) 56 and synaptophysin; chromogranin A and cytokeratin (CK) 7 staining were heterogeneously positive; thyroid transcription factor-1 (TTF-1) and CK5/6 were negative; Ki-67 nuclear positivity is 3% (Figure 1).

In the remaining lung tissue, NE cell hyperplasia was evident, with increased numbers of individual cell and small groups, confined to the bronchial or bronchiolar epithelium. Eleven tumorlets were also observed in recollected tissue from the surgical specimen, characterized by aggregates of NE cells with extension beyond the basement membrane and with less than 5 mm diameter. The cellular morphology and the immunohistochemical features were identical to the described carcinoid tumor.

There was no lung parenchyma disease and there was absence, in particular, of any inflammatory or fibrous lesions that could cause secondary NE cell hyperplasia.

The sampled lymph nodes did not show metastatic disease.

#### 3.1.4. Diagnosis

The final diagnosis was that of typical peripheral carcinoid tumor, associated with eleven tumorlets and DIPNECH.

#### 3.1.5. Follow-Up

The patient has been in clinical follow-up for 21 months and is so far free of disease.

### 3.2. Case 2

#### 3.2.1. Clinical Data

A female patient, 72 years old, presented with 6-month nonproductive cough. Physical examination was nonspecific. Pulmonary function tests showed a mild obstructive pattern. The patient was referred to our institution with CT-scan showing a well-defined pulmonary nodule which was 1.6 cm, located in the right middle lobe; there were also multiple bilateral nodules smaller than 5 mm; radiological signs of interstitial lung disease were not seen. These nodules could also be seen in a Positron Emitting Tomography (PET) scan. Bronchoscopy was normal. The patient then underwent surgical biopsy of the right middle lobe nodule.

#### 3.2.2. Macroscopy

Macroscopic examination of the surgical biopsy specimen showed a whitish and well-defined tumor, located close to the pleural surface, which was 1.6 cm. The pulmonary parenchyma had a normal macroscopic appearance. Total paraffin inclusion of the pulmonary resected tissue and of the pulmonary nodule was performed.

#### 3.2.3. Microscopy and Immunohistochemistry

**Figure 2:**
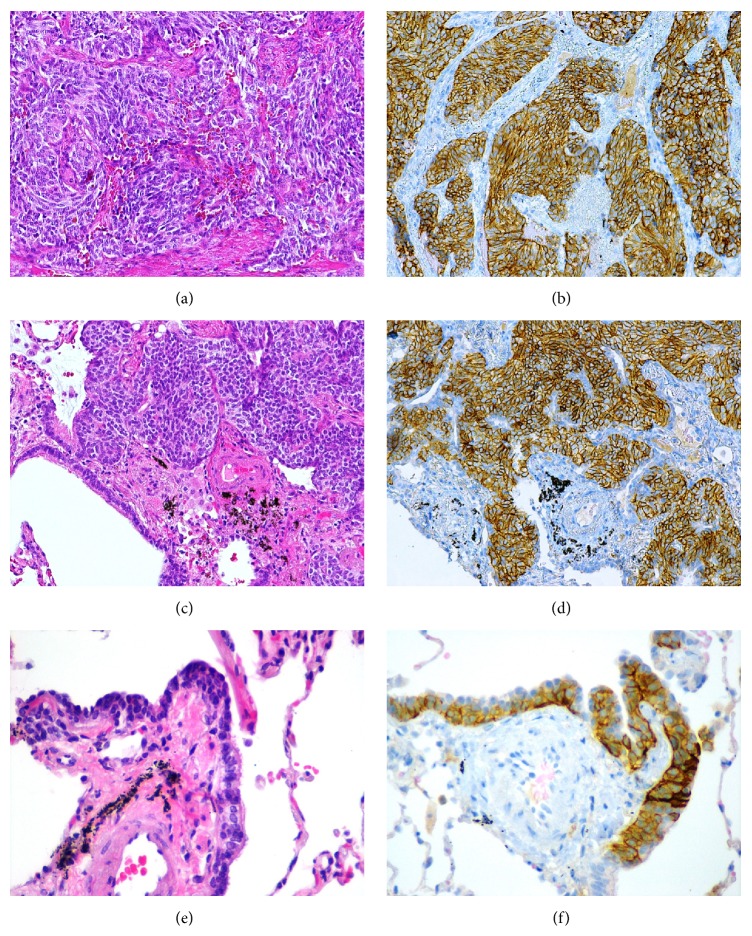
Case 2. (a) Carcinoid tumor. (b) Carcinoid with strong and diffuse expression of CD56. (c) Tumorlet. (d) Tumorlet also showing strong and diffuse expression of CD56. (e) DIPNECH. (f) Strong expression of CD56 in DIPNECH.

Microscopic study (Figure 2) showed that the tumor corresponded to a well-differentiated NE neoplasm, with expansive growth and an organoid or cordonal architectural pattern; the cells were elongated and had round or oval nuclei with “salt and pepper” chromatin and eosinophilic cytoplasm. As in the first case, there were less than 2 mitoses per 2 mm^2^ and necrosis was absent. Immunohistochemistry study showed diffuse staining for CD56, chromogranin A, synaptophysin, and CK7; TTF-1 staining was weakly positive and CK5/6 was negative; Ki-67 positivity was 2%.

The remaining lung tissue showed evident NE cell hyperplasia, with increased presence of individual cell and small cellular groups, confined to the bronchial or bronchiolar epithelium. Several tumorlets were also observed, characterized by aggregates of NE cells with extension beyond the basement membrane and less than 5 mm. The cellular morphology and the immunohistochemical features were identical to the carcinoid tumor previously described (Figure 2).

As in the first case, there was no significant lung parenchyma disease and there was absence, in particular, of any inflammatory or fibrous lesions that could cause secondary NE hyperplasia.

#### 3.2.4. Diagnosis

The final diagnosis was of typical peripheral carcinoid tumor, associated with several tumorlets and DIPNECH.

#### 3.2.5. Follow-Up

The patient is currently in clinical follow-up. The remaining nodules are stable in size and the patient is free of disease progression after a period of 18 months.

### 3.3. Case 3

#### 3.3.1. Clinical Data

An 80-year-old female patient presented, as in the second case, with chronic nonproductive cough with around 6-month duration. Physical examination was nonspecific and pulmonary functions tests were normal. The patient was referred to our institution with CT-scan showing a well-defined pulmonary nodule which is 1.4 cm, located in the right upper lobe; there were also multiple nodules smaller than 5 mm in the left lung; radiological signs of interstitial lung disease were not seen. These nodules could also be seen in a Positron Emitting Tomography (PET) scan. Bronchoscopy was normal. The patient then underwent surgical excision of the right upper lobe nodule.

#### 3.3.2. Macroscopy

Macroscopic examination of the surgical biopsy specimen showed a brownish and well-defined tumor, with elastic consistency, located near the pleural surface, which is 1.4 cm. The pulmonary parenchyma had unremarkable macroscopic appearance. Total paraffin inclusion was performed.

#### 3.3.3. Microscopy and Immunohistochemistry

**Figure 3:**
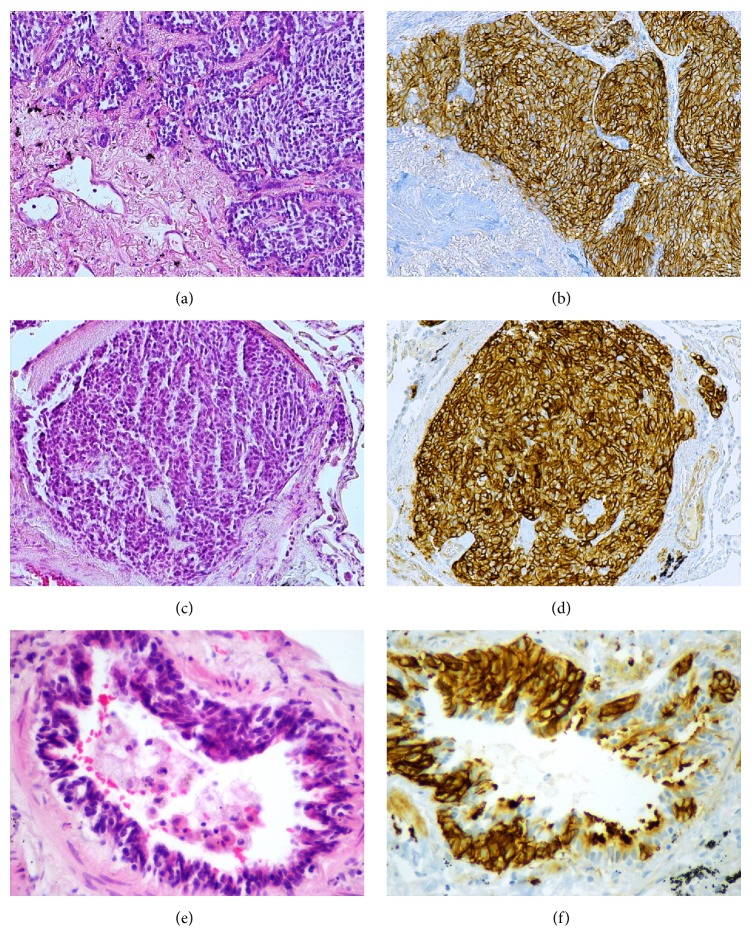
Case 3. (a) Carcinoid tumor; in the left lower corner central hyalinization can be seen. (b) Carcinoid with strong and diffuse expression of CD56. (c) Tumorlet. (d) Tumorlet also showing strong and diffuse expression of CD56. (e) DIPNECH. (f) Strong expression of CD56 in DIPNECH.

Microscopic study (Figure 3) showed that the tumor corresponded to a well-differentiated NE neoplasm, with expansive growth and showing an organoid and trabecular architectural pattern; the central region was hyalinized. The neoplastic cells were polygonal and elongated, smaller than in the two other cases, and had round or oval nuclei with “salt and pepper” chromatin and eosinophilic cytoplasm. Similar to the previous cases, there were less than 2 mitoses per 2 mm^2^ and necrosis was absent. Immunohistochemistry study showed diffuse staining for CD56, chromogranin A, synaptophysin, CK7, and TTF-1; CK5/6 was negative; Ki-67 positivity was 1%.

In this case the remaining lung tissue also showed evident NE cell hyperplasia, with increased numbers of individual cell and small groups, confined to the bronchial and bronchiolar epithelium. Several tumorlets were also observed, characterized by aggregates of NE cells with extension beyond the basement membrane and less than 5 mm in diameter.

There was also in this case no significant lung parenchyma disease and there was absence, in particular, of any inflammatory or fibrous lesions that could cause secondary NE hyperplasia.

#### 3.3.4. Diagnosis

The final report described a typical peripheral carcinoid tumor, associated with several tumorlets and DIPNECH.

#### 3.3.5. Follow-Up

The patient is currently in clinical follow-up. The remaining nodules (in the left lung) are stable in size and the patient is free of disease progression after a period of 15 months.

## 4. Discussion

DIPNECH (not associated with underlying lung disease) is thought to be precursor of pulmonary tumorlets and carcinoid tumors [4]. It is rare to see this spectrum of NE proliferation in the same specimen, with these being the only 3 cases diagnosed in our department in the last 25 years. Up until recently, only a small number of well-documented cases and small reviews were published in the English literature [1, 6–9]. However, a recent published review [10] comprising 199 DIPNECH cases (169 from the literature and 30 from the authors) established the coexistence of carcinoid tumors with DIPNECH in the same specimen to be around 50%. DIPNECH has never been described in association with the more aggressive neuroendocrine carcinomas, such as large cell and small cell neuroendocrine carcinomas [4, 10].

The majority of patients appear to be nonsmoker women, in their fifth or sixth decade [4, 10]; our cases were all women with an average age of 72 years, a little over that recognized in the literature. Symptomatic patients can show dry cough and/or dyspnea with very slow worsening over several years [1, 4, 6–8, 10]. Asymptomatic patients with pulmonary nodules detected in radiological evaluation for other reasons have also been reported [1, 4, 6–8, 10]. Physical examination was usually nonspecific and pulmonary function tests might be normal, showing an obstructive pattern (present in one of our cases), restrictive pattern, or mixed obstructive/restrictive pattern [4, 10].

Plain chest radiographs were reported as normal or showed at least one nodule [1, 6–8]. HRCT scans of the thorax revealed one or more nodules, corresponding to tumorlets or carcinoid tumors [1, 6–8, 10]; HRCT can also show a mosaic pattern of air trapping with thickened bronchial and bronchiolar walls, bronchiectasis, or ground-glass attenuation [4, 6–8, 11]. In our cases the nodules corresponding to the carcinoid tumors were evident, as were the smaller nodules corresponding to the tumorlets in one of the cases; no other alterations of the lung parenchyma were described before pathology study.

Clinically and on imaging, DIPNECH can be indistinguishable from other diffuse lung diseases with identical signs and symptoms and the diagnosis is usually made through lung biopsy. However, a HRCT showing mosaic attenuation associated with multiple small nodules has been described as being strongly suggestive of DIPNECH diagnosis [4, 8, 11].

DIPNECH is invisible to the naked eye. Macroscopic examination can however sometimes detect little grey-white nodules corresponding to tumorlets or small carcinoids [4]. Large carcinoid tumors, as in our cases, can be readily identified as firm, homogeneous, grey-white or brownish, and well-defined nodules [4].

Microscopic examination of a DIPNECH case usually reveals diffuse proliferation of NE cells, ranging from a small increase in the number of individual bronchial/bronchiolar NE cells to the formation of groups confined to the bronchial or bronchiolar walls; larger lesions can bulge into lumina [4]. The bronchiolar wall can be fibrotically thickened, which can lead to bronchiolar occlusion, together with the NE cells proliferation [7]. However, inflammatory or fibrotic lesions of the lung parenchyma that could cause secondary NE cell hyperplasia are absent [4]. DIPNECH, as in our cases, can be associated with NE cell nodules extending beyond the basement membrane, which, when present, warrants the designation of tumorlet, when less than 5 mm in diameter; larger lesions are termed carcinoid tumors [4].

Histopathological observation of DIPNECH must then be distinguished from reactive NE proliferations secondary to lung parenchyma diseases; the presence of causative pathology and absence of carcinoid tumors is essential for this distinction [4]. A recent published study [12] reviewed 70 consecutive lung resection cases with multifocal NE cell proliferation, including NE cell hyperplasia and/or more than 1 tumorlet; the presence of multifocal NE cell hyperplasia (defined by the presence of 5 or more NE cells, singly or in clusters located within the basement membrane of the bronchiolar epithelium of at least 3 bronchioles) combined with 3 or more tumorlets was proposed as the minimum criteria for the pathologic diagnosis of DIPNECH. The authors of the study recognized, however, that a larger series of consecutive surgical specimens and autopsies was needed to verify more accurately the proposed criteria [12]. Perhaps these criteria will help in clarifying the relationship of DIPNECH with the increase of NE cells that can be seen in the parenchyma immediately adjacent to many carcinoids tumors; although still unclear, it is limited, in the latter, to the immediate vicinity of the tumor [4, 13].

Genetic studies [14] of NE lung lesions have shown that carcinoid and large cell/small cell neuroendocrine carcinomas are biologically different and do not comprise one spectrum of NE lung disease; this supports DIPNECH as the precursor of carcinoids and suggests a different and yet unidentified origin for high grade NE carcinomas. Carcinoids were shown to have allelic imbalance of 11q13 region, an alteration rare or absent in tumorlets and that may be the trigger of tumor development [15]. DIPNECH has also been associated occasionally with multiple neuroendocrine neoplasia type 1 (MEN1) [4].

There is currently no predictive histological or genetic data for DIPNECH. Surgical excision of the dominant lesion is typically advised [1, 4]. As previously reported [1, 4, 6, 7, 10], the presented cases reinforce a prolonged clinical course, with stable (or slowly progressive) disease over many years. The associated carcinoid tumors are also reported as indolent and only rarely atypical features or extrapulmonary metastatic diseases have been described [10]. Occasionally, patients have developed a progressive constrictive bronchiolitis; in these cases, there is no evidence to show that somatostatin analogues (like octreotide) alter the course of the disease [10]. Treatments for the obstructive symptoms with bronchodilators and corticosteroids (inhaled or systemic) or lung transplantation are the current options [1, 4, 6, 7, 10].

## Figures and Tables

**Table 1:** Immunohistochemistry: antibodies used on this study.

Antigen	Clone	Dilution	Antigen retrieval	Source	Detection system
CD56	MRQ-42	Ready to use	Ultra CC1	Rabbit	Ultraview DAB Ventana
Chromogranin A	LK2H10	Ready to use	Ultra CC1	Mouse	Ultraview DAB Ventana
Synaptophysin	MRQ-40	Ready to use	Ultra CC1	Rabbit	Ultraview DAB Ventana
CK5/6	D5/16B4	Ready to use	Ultra CC1	Mouse	Ultraview DAB Ventana
CK7	SP52	Ready to use	Ultra CC1	Rabbit	Ultraview DAB Ventana
TTF-1	SP141	Ready to use	Ultra CC1	Rabbit	Ultraview DAB Ventana
Ki-67	30-9	Ready to use	Ultra CC1	Rabbit	Ultraview DAB Ventana

CC1: cell conditioning 1; DAB: diaminobenzidine.
